# Impact of Social Cognition on the Self-Rated Health of the Elderly and Its Mechanisms: Evidence From China’s Comprehensive Social Survey

**DOI:** 10.3389/fpsyg.2021.737081

**Published:** 2022-01-17

**Authors:** Yuan Liu, Yuqun Hu, Yan Nan

**Affiliations:** School of Public Policy and Administration, Xi’an Jiaotong University, Xi’an, China

**Keywords:** social cognition, social attitude, class identity, perception of social relations, self- rated health, social engagement

## Abstract

Whether and how the differentiated social cognition of the elderly affects their self-rated health has not been deeply discussed. Based on social cognition theory and Chinese situation, this study constructs the social cognitive dimension of Chinese elderly including social attitude, class identity and perception of social relations. Using the data from Chinese General Social Survey in 2017, this study screens out 1,728 elderly people aged 60 and over, and discusses the impact mechanism of social cognition on self-rated health of Chinese elderly people aged 60 and over by the construction of structural equation model and mediation effect test method. The results show that social attitude, class identity and perceptions of social relationships have significantly positive effects on the self-rated health levels of elderly individuals. Among them, the path coefficient of social attitude to self-rated health was 0.049, the path coefficient of class identity to self-rated health was 0.171, and the path coefficient of social relationship perception to self-rated health was 0.248; both class identity and perception of social relationship have significant mediating effects on elderly self-rated health through social engagement. This study shows that social cognition rarely studied in existing literature has significant effects on the self-rated health of older adults, providing fruitful insights for enhancing the self-rated health level of elderly individuals.

## Introduction

Self-rated health refers to an individual’s perceived health status, which is called perceived health, self-assessed health and so on (Pan and Wu, 2018). It is a sequential choice of “very good, good, average, poor and poor” made by the respondent after comparing with the reference group or the ideal health status of the respondent (Liu, 2009; Qi, 2014). Self-rated health is an important predictor of morbidity and mortality. It can positively predict the incidence rates of various diseases and mortality risk among elderly individuals, which is very important for observing overall health of elderly individuals and development of overall health in society (Anne et al., 2009; Pan and Wu, 2018). As a comprehensive evaluation of their own past, present and future objective physical conditions and subjective mental health levels, self-rated health correlates strongly with objective health status and can reflect the actual health status of elderly individuals. Therefore, self-rated health has become a common index for evaluating the health status of the middle-aged and elderly in social surveys, and one of the international general health measurement methods (Fang et al., 2003), which has been widely used in health research in the United States, Canada and many other industrialized countries (Paskett et al., 2010; Jia et al., 2016). There are many factors affecting self-rated health, but there is no unified conclusion at present. Sociodemographic factors such as age, education level and marital status have been considered as the main factors affecting self-rated health (Zhang and Yu, 2019). Because self-rated health is based on the subjective perceptions and evaluations of individuals, it depends on the pathological burden on the body as well as the social and cultural background of the individual. Self-rated health is less affected by the body’s health status among elderly individuals than among young individuals. Therefore, differences in the social cognitive levels of elderly individuals may affect their self-rated health (Idler and Cartwright, 2018).

At present, there are few references discussing how social cognition influences Chinese elderly Individuals’ self-rated health. First, existing studies take social cognition as a dimension of social capital and discuss its influence on self-rated health (Xue and Cheng, 2012), but rarely discuss it from the unique perspective of social cognition. Second, most of the studies simply equate “self-rated health” with “health” measurement, without clarifying the value of self-rated health itself. Third, domestic research is mostly based on data of a certain province or county (Mi et al., 2016), and there is little research based on the national data, so the results are not of great general significance. Fourth, current research mostly uses the path test method of multiple regression, which can not only avoid the complex path in the estimation process, but also cause deviation in the estimation of intermediary variables, which affects the quality of the research. In order to further explore whether and how social cognition affects the self-rated health of elderly groups. In this study, we used the 2017 China General Social Survey (CGSS) data to analyze the impact of social cognition on the self-rated health of Chinese individuals aged 60 or older by using structural equation modeling. At the same time, social engagement is an important part of elderly individuals’ lifestyles, and can greatly promote the physical and mental health of elderly individuals and reduce the risk of death (Huang, 2017; Yang and Wang, 2020). We further introduced social engagement variables in this study to verify the mediating effect of social engagement between social cognition and self-rated health. Our findings have implications for improving the self-rated health of elderly individuals, reducing their risk of death, and improving their well-being and quality of life in old age.

## Literature Review and Research Hypothesis

### Social Cognition and Self-Rated Health

In 1985, Bandura proposed the theory of social cognition, which emphasizes that human activities are determined by the interaction of individual behavior, individual cognition and the environment in which an individual lives (Bandura, 1985). Social cognition is a process that includes processing information and reasoning inductively about past experiences and relevant cognitive clues, and it is the basis for individual behavior. The development of social cognitive theory has been rich and includes social equity theory and social norms theory. Man (2017) divided social cognition into three dimensions, i.e., social attitudes, class identity and public service satisfaction, and analyzed their impacts on Chinese residents’ self-rated health. Li et al. (2020) and others divided social cognition into social trust, social justice and class identity to analyze the entrepreneurship of urban returnees. Pan and Wu (2018) focused on the micro level of individual social capital cognition and divided this cognition into three dimensions, i.e., social trust, perception of social help and perceptions of reciprocity, to explore its impact on self-rated health of elderly individuals in rural China. Based on previous studies, this study divides social cognition into three dimensions: social attitude, class identity and social relationship perception. Among them, Social attitude is a state of psychological preparation, which is the individual’s evaluation and behavior tendency to whole society or a certain object of society (Tang, 2000), This study mainly discusses the perception and evaluation of the elderly group’s trust and fairness to whole society; class identity is an individual’s subjective judgment of his position in social class and socio-economic status based on objective conditions and subjective perception (Tonglet et al., 2004). Perception of social relations is the perception of the elderly about the relationship between themselves and the people around them, which mainly reflects the sense of help and reciprocity of the people around them. Next, this study will further explore the effects of social attitude, class identity and social relationship perception on the self-rated health of the elderly.

**FIGURE 1:**
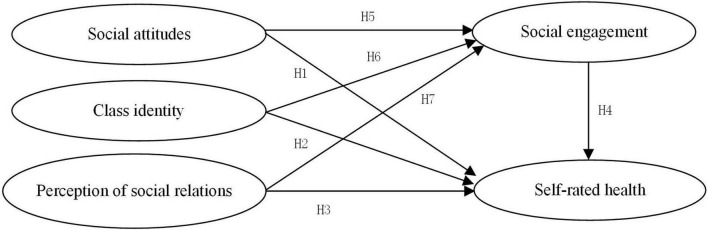
Path hypothesis model.

Sun used CGSS data from 2008 to prove that education has a significant impact on the perceived health of urban and rural Chinese residents through social attitudes and individual cognitive levels (Sun and Li, 2013). Based on rural survey data from Hubei and Henan, Xue and Cheng (2012) found that social trust is an important factor affecting the health of elderly individuals in rural areas. As an individual’s subjective evaluation of his or her own objective social stratification, class identity has stronger predictive power than the individual’s objective class. Residents’ different class self-perceptions lead to different levels of social cognition and environmental dependence and, in turn, different levels of self-perceived health and individual health self-evaluations (Guo, 2016). Shen et al. (2014) explored the influence of social capital cognition on the self-rated health status of elderly Chinese people and found that perceived helpfulness at the individual level correlated significantly with self-rated health. Based on this, we assume that the more positive the social attitudes of elderly individuals, the higher their self-rated health levels (see Figure 1, H1); the higher their class identity levels, the higher their self-rated health levels (see Figure 1, H2); and the better their perceived social relationships, the higher their self-rated health levels (see Figure 1, H3).

### Social Engagement and Self-Rated Health

At present, there is no unified definition of the social activities of elderly individuals. Engel divided the social activities of elderly individuals into seven types: participating in social production, participating in various skills training and learning courses, participating in various charity activities, participating in various sports activities, participating in various volunteer activities, and participating in various organizations or clubs, including religious organizations or clubs. Yang (1995) and Du and Wang (2011) concluded that regardless of the form through which elderly individuals keep in contact with society, the activities used are social engagement activities. Some scholars have proposed that helping to take care of their grandchildren, participating in housework, reading books and newspapers at home, watching TV, surfing the Internet and engaging in other family recreational activities are also social activities for elderly individuals (Liu, 2006; Yang and Wang, 2021). As for social engagement, there is no unified conclusion in academic circles. Levasseur et al. (2010) collected and sorted out relevant studies from 1980 to 2009 and screened 43 definitions of social engagement. Through content analysis from seven dimensions. They proposed that social engagement can be defined as an activity in which individuals participate and are able to interact with others in a society or community. This comprehensive definition points out the most essential characteristics of social engagement. Therefore, referring to the definition of Levasseur, we believe that the social engagement of the elderly is an activity in which the elderly voluntarily participate and can interact with others in the society or community.

International research on the influence of social engagement on the self-evaluated health of elderly people is more unified. Iwasaki et al. (2001), Sasidharan et al. (2006), and Lee et al. (2008) argued that the more frequently elderly individuals participate in social activities, the better their self-perceived physical health status. This conclusion is basically consistent with the views of the Chinese scholars Yang and Hu (2016). Rowe et al. (2009) showed that intense participation in social activities can effectively buffer the adverse effects of work stress on health, which is an important factor for guaranteeing the health of elderly individuals. Carlson et al.’s (2008) study found that participation in high-intensity volunteer activities that require certain cognitive abilities can delay declines in cognitive ability among elderly individuals. Social activities can provide elderly individuals with different types of social roles, help them realize their own value, and provide a sense of participation and satisfaction. Therefore, we hypothesize that the higher the level of social engagement among elderly individuals, the higher their self-rated health levels (see Figure 1, H4).

### Social Cognition and Social Engagement

In real life, people have limited rationality, and emotional and subjective perceptions are often more important than objective indicators (Esaiasson, 2010; Sun et al., 2019). Social cognition, emotions, values and so on affect or even control individual behavior choices. Social attitudes include perceptions of social trust and social justice, among which personal perceptions of social trust have a substantial impact on personal access to information and social networks (Guinnane, 2005). Xu and Shi (2016) found that social trust has a significantly positive impact on the social engagement of urban migrants. The higher is the degree of social trust, the more active is the social engagement of migrants. Huo et al. (2017) found that there is a significantly positive correlation between trust and participation in social networks by studying the influence of user trust on participation in mobile social networks. As a part of social attitudes, perceptions of social justice are the subjective psychological feelings experienced when people evaluate social justice. Based on CGSS 2015 data, Sun (2019) found that perceptions of social justice had a significantly positive effect on the integration of the rural migrant population into cities and their citizenization. Class identity is an individual’s perception of his or her position in the social class structure (Gao, 2013). Many studies have shown that people with lower class identities are more likely to exhibit strong prosocial intentions (Piff et al., 2010). Social cognition theory holds that this is a socially adaptive response of people of a lower class to natural and social threats. Perceptions of social relationships mainly refer to the individual’s perceptions of his or her interactions with others, which reflect not only the social network resources available but also enthusiasm for social engagement within those networks. Based on this, in this study, we hypothesize that older people with a more positive social attitude have a higher degree of social engagement (see Figure 1, H5), older people with a lower class identity have a higher degree of social engagement (see Figure 1, H6), and elderly individuals who are more satisfied with their perceptions of their social relationships have a higher degree of social engagement (see Figure 1, H7).

## Materials and Methods

### Data Sources and Sample Selection

The data used in this study is from the 2017 CGSS. The CGSS is the longest-running national, comprehensive and continuous academic investigation project in China, and the data of 2017 are the most recent survey data from this project to be released. The CGSS adopts multilevel sampling and covers all provincial administrative units in mainland China. This project uses a face-to-face questionnaire to administer a continuous cross-sectional survey and systematically and comprehensively collects data on multiple levels: society, community, family and individual. It covers rural and urban populations over 18 years of age. The CGSS in 2017 included 12,582 observations. As this study is focused on elderly individuals aged 60 or above, after the age group was selected and observations that were missing data for key variables were excluded, 1,728 observations were included in the analysis.

### Variable Measurement

According to Bollen (1989), each dimension in a structural equation model generally needs at least three observed variables to capture its content, and collinearity should be avoided. A total of 18 observed variables were selected for the effective observations (see Table 1), and these variables reflect five potential dimensions: social attitudes, class identity, perceptions of social relationships, social engagement and self-rated health. The 18 items are either continuous variables or ordered variables and meet the requirements for structural equation model analysis. The specific questions and answers to the 18 observation variables are shown below.

**TABLE 1:** Distribution and coding of variable items.

Index	Data code	Item	Min	Max	Code
Social attitudes	A33	Generally speaking, do you agree that most people in this society can be trusted?	1	5	X1
	C10	Do you think people will take advantage of you if they have a chance, or will they try to be fair?	1	4	X2
	C11	Generally speaking, do you think people are always trustworthy, or do you have to be very careful when dealing with people?	1	4	X3
Class identity	A43b	What level of society do you think you were in 10 years ago?	1	5	X4
	A43c	What level of society do you think you will be in 10 years from now?	1	5	X5
	A43a	In general, what level of society do you think you are in?	1	5	X6
Perception of social relations	C91	How often in the past 4 weeks have you felt a lack of company?	1	4	X7
	C92	How often in the past 4 weeks have you felt isolated from others?	1	4	X8
	C93	How often have you felt left out in the past 4 weeks?	1	4	X9
Social engagement	A3005	In the past year, did you often participate in cultural activities in your spare time, such as concerts, performances and exhibitions?	1	5	X10
	A3012	In the past year, did you often surf the Internet in your spare time?	1	5	X11
	A3009	In the past year, did you often take part in physical exercise in your spare time?	1	5	X12
	C51	How frequently did you participate in activities organized by leisure groups, sports groups or cultural groups in the past 12 months?	1	5	X13
	C52	In the past 12 months, how often did you participate in activities organized by political parties, political groups or political associations?	1	5	X14
	A3007	In the past year, did you often get together with your friends in your spare time?	1	5	X15
Self-rated health	A15	What do you think of your current health?	1	5	X16
	A16	How often have health problems affected your work or other daily activities in the past 4 weeks?	1	5	X17
	A17	How often did you feel depressed in the past 4 weeks?	1	5	X18

(1) Social attitudes. This study mainly discusses the perception and evaluation of the elderly group’s trust and fairness to whole society, and mainly reflects their social attitude from the elderly group’s perception of social trust and social fairness. In the CGSS 2017 questionnaire, we use A33 to ask “Generally speaking, do you agree that most people in this society can be trusted?” which answers are “strongly disagree,” “somewhat disagree,” “hardly agree disagree,” “somewhat agree” and “strongly agree” respectively, and the above answers are assigned 1–5 points in order. We use C10 to ask “Do you think people will take advantage of you if they have a chance, or will they try to be fair?” which answers are “always trying to take advantage of you” “mostly trying to take advantage of you” “mostly trying to be fair” “always trying to be fair,” and the above answers are assigned 1–4 points in order. We use C11 to ask “Generally speaking, do you think people are always trustworthy, or do you have to be very careful when dealing with people?” which answers are “You can’t be too careful when dealing with people,” “usually, you can’t be too careful when dealing with people,” “people can usually be trusted,” “people can always be trusted,” and the above answers are assigned 1–4 points in order. The higher the score, the more positive the social attitude of the older group.

(2) Class identity. Self-evaluation identification is one of the effective methods to measure subjective class identity. CGSS 2017 set up special class identity survey projects, A43 first tip “in our society, some people in upper class, some people in lower class,” by default the objective existence of social class, and then presented the intuitive social class is demonstrated, where “1” represents the bottom layer, “10” represents the top layer, and the numbers 1–10 increase in turn. Then we use A43b to ask “What level of society do you think you were in 10 years ago?” A43c to ask “What level of society do you think you will be in 10 years from now?” and A43a asking “In general, what level of society do you think you are In?” the questionnaire used 1–10 points to represent the subjective class identity of the respondents, and we further processed 1–10 points into 1–5 points. The higher the score is, the higher the subjective class identity of the elderly group is.

(3) Perception of social relations. The perception of social relations in this study is mainly the perception of the elderly on the relationship between themselves and the people around them, so as to reflect the sense of help and reciprocity of the people around them. In the CGSS 2017 questionnaire, we use C91 to aske “How often in the past 4 weeks have you felt a lack of company?” C92 to aske “How often in the past 4 weeks have you felt isolated from others?” and C93 to ask “How often have you felt left out in the past 4 weeks?” The answers are “often,” “sometimes,” “rarely,” and “never.” We assign 1–5 points to the answers in turn. The higher the score, the better the perception of social relations of the elderly group.

(4) Social engagement. This study defines that the social engagement of the elderly is an activity in which the elderly voluntarily participate and can interact with others in the society or community. In the CGSS 2017 questionnaire, firstly, we mainly reflected the elderly’s participation in activities in their spare time through the following four questions. We use A3005 to asked “In the past year, did you often participate in cultural activities in your spare time, such as concerts, performances and exhibitions?” A3012 to ask “In the past year, did you often surf the Internet in your spare time?” A3009 to ask “In the past year, did you often take part in physical exercise in your spare time?” and A3007 to ask “In the past year, did you often get together with your friends in your spare time?” The answers are “Never,” “several times a year or less,” “several times a month,” “several times a week” and “every day.” We assign 1–5 points to the answers in turn. The higher the score, the more active the elderly participate in social activities in their free time. Secondly, we mainly reflect the participation of the elderly in group activities through the following two questions. We use C51 to ask “How frequently did you participate in activities organized by leisure groups, sports groups or cultural groups in the past 12 months?” and C52 to ask “In the past 12 months, how often did you participate in activities organized by political parties, political groups or political associations?” The answers are “Never participated,” “participated once last year,” “participated several times last year,” “1–3 times a month,” “1 or more times a week.” We assign 1–5 points to the answers in turn. The higher the score, the more active the elderly participate in group activities.

(5) Self-rated health. This study mainly refers to the perceived evaluation of the elderly on their own physical and mental health. In the CGSS 2017 questionnaire, we use A15 to ask “What do you think of your current health?” The answers are “Very unhealthy” “relatively unhealthy” “average” “relatively healthy” “very healthy,” and the above answers are assigned 1–5 points in order. The higher the score, the better the overall health of the elderly. We use A16 to ask “How often have health problems affected your work or other daily activities in the past 4 weeks?” The answers are “Always” “often” “sometimes” “rarely” “never,” and the above answers are assigned 1–5 points in order. The higher the score, the better the physical health of the elderly. We use A17 to ask “How often did you feel depressed in the past 4 weeks?” The answers are “Always” “often” “sometimes” “rarely” “never,” and the above answers are assigned 1–5 points in order. The higher the score, the better the mental health of the elderly.

The selected demographic variables included gender, age, education level, personal annual income, marital status and household registration status. The purpose of collecting these variables was to observe the sample distribution and test its representativeness.

### Statistical Analysis Method

SPSS 21.0 and Amos 24.0 were used to analyze the data, and a structural equation model was used to analyze the mediating effect of social engagement on self-rated health. First, this paper analyzed correlations among five variables: social attitude, class identity, perceptions of social relationships, social engagement and self-rated health. Second, a structural equation model was used to construct the paths of influence and the structural relationships among the variables to obtain an intermediary structural model and to analyze the mediating effects. Finally, the bootstrap method was used to obtain the results of the significance tests for the mediating effect.

### Descriptive Statistics

The distribution of the basic characteristics of the sample population is as follows. A total of 846 observations were men (49%), and 882 were women (51%). A total of 901 people (52.1%) had an education level below primary school, 445 people (25.8%) had a junior middle school education level, 193 people (11.2%) had a senior high school education level, 132 people (7.6%) had a college or technical school education level, and 57 people (3.3%) had an undergraduate education level or above. As the range of annual incomes among elderly individuals is large, a logarithmic transformation was used. According to the histogram of logarithmic frequency distribution, the distribution basically followed a normal distribution. There were 60 unmarried elderly people (3.5%), 1,274 married people (73.7%), 28 divorced people (1.6%), and 366 widowed people (21.2%); 695 urban elderly people (40.2%) and 1,033 rural elderly people (59.8%) were included. Through this investigation of the distribution of the basic characteristics of the sample population, we found that the selected elderly population sample had good representativeness.

### Reliability and Validity Testing

Reliability refers to the degree of consistency or stability in the measurement results; because the items in the questionnaire in this study were not measured repeatedly, the indicators reflecting internal consistency were mainly used to measure the validity of data. After importing all relevant items into SPSS 21.0 for analysis, Cronbach’s alpha coefficient was found to be 0.762. After introducing the relevant items of social attitude, class identity, perception of social relations, social engagement and self-rated health into SPSS 21.0 for analysis, it was found that Cronbach’s alpha coefficients were 0.732, 0.824, 0.881, 0.641, and 0.764, respectively. The larger the Cronbach’s alpha coefficient, the higher the reliability of the measure and the higher the internal consistency of the questionnaire, indicating that the data can be used for correlation analysis.

Validity refers to the effectiveness of the measurements; that is, the more consistent the measurement results are with content to be investigated, the higher the validity of the measures; less consistent measurement results indicate lower validity. After importing all relevant items into SPSS 21.0, KMO and Bartlett sphericity tests were carried out. The KMO value was 0.718, the Bartlett sphericity test chi-squared statistic was 15277.814, and the *p*-value was 0.000 (< 0.05), indicating that there were correlations among the variables and that the data was suitable for factor analysis.

Internal consistency was measured to test the reliability of each dimension. Following Hou and Chen (2014) and Chen and Lu (2021) and others, the corrected item total correlation (CITC) was calculated, and observed variables with poor reliability were deleted. The higher the CITC value, the higher the discrimination of the corresponding items. If the CITC value is greater than 0.3, the variable is acceptable. The test results are shown in Table 2, which shows that the questionnaire data structure was valid and that the data could be analyzed accordingly.

**TABLE 2:** Reliability and validity tests of the variable indexes.

Index	Code	CITC	Index	Code	CITC
Social attitudes	X1	0.307	Social engagement	X10	0.436
	X2	0.740		X11	0.403
	X3	0.730		X12	0.402
Class identity	X4	0.565		X13	0.473
	X5	0.686		X14	0.323
	X6	0.801		X15	0.335
Perception of social relations	X7	0.684	Self-rated health	X16	0.626
	X8	0.810		X17	0.671
	X9	0.833		X18	0.507

### Ethics Statement

In accordance with the local legislation and institutional requirements, the study on human participants does not require ethical review and approval. The participants provided their written informed consent to participate in this study.

## Results and Analysis

### Fitness Analysis of the Structural Equation Model

**FIGURE 2:**
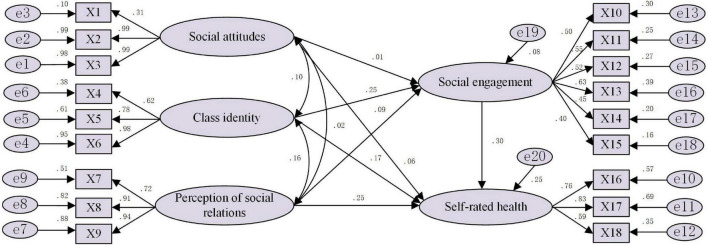
Path estimation results for the structural equation model.

In this paper, we mainly use 11 major fitting indexes to test the fit of the constructed structural equation model from the three dimensions of absolute fit, value-added fit and simple fit. By comparing the output values from and the reference values for the fitting indexes, we can evaluate the degree of fit between the model and the sample data. We used Amos 24.0 software to construct structural equations, conduct path analysis on the data, and obtain the results of the fitness test of the research model (see Table 3) and the standardized path coefficient of the research model (see Figure 2). The fitting index represents how well the model fits the survey data. For this model, the chi-squared degrees of freedom ratio was 4.416 (< 5) (Chen and Lu, 2021), GFI was 0.968 (> 0.9), and IFI and CFI were 0.974, both higher than 0.9. In addition, the fit was good: the PNFI was 0.789 (> 0.5), RMSEA was 0.043 (< 0.08), and TLI was 0.968 (> 0.9). The above data showed that the modified parameters of each fitting index were in line with the accepted critical values, which indicateed that the constructed structural equation model fit the data well overall and could be used for hypothesis development.

**TABLE 3:** Results of the model fitness tests.

Classification of fit index		Model fitting value	Reference value	Result
Absolute fit index	GFI	0.968	>0.9	Acceptable
	AGFI	0.956	>0.9	Acceptable
	RMSEA	0.043	<0.08	Acceptable
Value-added fit index	NFI	0.966	>0.9	Acceptable
	RFI	0.959	>0.8	Acceptable
	IFI	0.974	>0.9	Acceptable
	TLI	0.968	>0.9	Acceptable
	CFI	0.974	>0.9	Acceptable
Parsimony adaptation index	PCFI	0.796	>0.5	Acceptable
	PNFI	0.789	>0.5	Acceptable
	CMIN/DF	4.416	<5.0	Acceptable

### Research Hypothesis Testing

#### Estimation Results of the Structural Equation Model

With the help of Amos software, we obtained the structural equation model (see Figure 2) and path coefficients (see Table 4). The results showed that in the elderly group, the path coefficient of social attitude to self-rated health was 0.049, reaching a significance level of 10%; the path coefficient of class identity to self-rated health was 0.171, reaching a significance level of 1%; and the path coefficient of social relationship perception to self-rated health was 0.248, reaching a significance level of 1%. The more positive the social cognition of the elderly, the higher their self-rated health level. The path coefficient of social engagement to self-rated health was 0.303, reaching a significant level of 1%, which indicates that the higher the degree of social engagement, the higher the self-rated health level of the elderly. Social attitude had no significant effect on the social engagement of the elderly; by contrast, the path coefficient of class identity on the social engagement of the elderly was 0.253, and the path coefficient of social relationship perception on the social engagement of the elderly was 0.085. These results show that in the social cognition of the elderly, their social attitude of the elderly does not affect the degree of their social engagement, which may be related to the particularity of social engagement. The elderly mostly socialize with familiar people. The higher the level of class identity, the more satisfied the perception of social relations, the higher the degree of social engagement of the elderly.

**TABLE 4:** Estimation results for the path coefficients.

Path explanation			Non-standardized path coefficient	S.E.	C.R.	*P*	Standardized path coefficient
Social engagement	←	Social attitudes	0.006	0.015	0.373	–	0.011
Social engagement	←	Class identity	0.114	0.014	7.978	***	0.253
Social engagement	←	Perception of social relations	0.039	0.014	2.841	**	0.085
Self-rated health	←	Social attitudes	0.053	0.027	1.974	*	0.049
Self-rated health	←	Class identity	0.160	0.026	6.215	***	0.171
Self-rated health	←	Perception of social relations	0.237	0.026	9.256	***	0.248
Self-rated health	←	Social engagement	0.635	0.073	8.691	***	0.303
X1	←	Social attitudes	0.407	0.030	13.390	***	0.309
X2	←	Social attitudes	0.970	0.013	74.498	***	0.994
X3	←	Social attitudes	1.000				0.990
X4	←	Class identity	0.631	0.024	26.019	***	0.616
X5	←	Class identity	0.848	0.026	33.146	***	0.783
X6	←	Class identity	1.000				0.976
X7	←	Perception of social relations	0.860	0.024	36.327	***	0.717
X8	←	Perception of social relations	0.941	0.018	50.952	***	0.905
X9	←	Perception of social relations	1.000				0.938
X10	←	Social engagement	1.000				0.548
X11	←	Social engagement	1.940	0.136	14.260	***	0.504
X12	←	Social engagement	2.227	0.153	14.515	***	0.518
X13	←	Social engagement	1.808	0.113	15.989	***	0.627
X14	←	Social engagement	0.858	0.065	13.240	***	0.450
X15	←	Social engagement	1.081	0.090	12.072	***	0.396
X16	←	Self-rated health	1.000				0.756
X17	←	Self-rated health	1.243	0.049	25.540	***	0.832
X18	←	Self-rated health	0.746	0.034	21.913	***	0.592

*, **, *** Indicate that the statistical results are significant at the confidence level of 10%, 5% and 1% respectively.

The measurement model reflects the relationship between the observed variables and potential variables. The relationships reflected by the measurement model in Table 4 can be summarized as follows: (1) The standardized path coefficient between social justice and social attitude was 0.994, and the standardized path coefficient between social trust and social attitude was 0.990, which indicates that the higher the level of social trust and social justice perceived by the elderly, the more positive the social attitude of the elderly will be. (2) Among the three indicators reflecting the subjective class identity of the elderly, the indicators that had the greatest impact on class identity were the perceived social level at present and the perceived social level 10 years later. The standardized path coefficients of these two indicators were 0.976, and 0.783, respectively. (3) Among the three indicators reflecting the elderly’s perception of social relations, being ignored by others and being isolated by others were the two most influential indicators, with standardized path coefficients of 0.905, and 0.938, respectively. (4) Among the six indicators reflecting the social engagement of the elderly, the influences of group activity participation, cultural activity participation, physical exercise, Internet access, political community activity participation and friend gathering on the social engagement of the elderly successively decreased, with standardized path coefficients of 0.627, 0.548, 0.518, 0.504, 0.450, and 0.396, respectively. (5) Among the three indicators reflecting self-rated health of the elderly, the perceived health status of the elderly had the greatest impact on their self-rated health, with a standardized path coefficient of 0.832.

Because the coefficients of each path were significant, we verified the hypotheses. The paths of “social attitudes—self-rated health, class identity—self-rated health, perception of social relations—self-rated health, social engagement—self-rated health, class identity—social engagement, perception of social relations—social engagement” were all significant at *p* < 0.1. Therefore, the H1, H2, H3, H4, and H7 path tests were all significant, whereas H6 was significant but opposite in direction compared to the prediction. In contrast, the path of “social attitude—social engagement” was p > 0.1, and the H5 path test was not significant (see Table 5).

**TABLE 5:** Research hypothesis test results.

Path explanation			Standardized path coefficient	S.E.	C.R	*P*	Corresponding hypothesis
Social engagement	←	Social attitudes	0.011	0.015	0.373	–	H1
Social engagement	←	Class identity	0.253	0.014	7.978	***	H2
Social engagement	←	Perception of social relations	0.085	0.014	2.841	**	H3
Self-rated health	←	Social attitudes	0.049	0.027	1.974	*	H4
Self-rated health	←	Class identity	0.171	0.026	6.251	***	H5
Self-rated health	←	Perception of social relations	0.248	0.026	9.256	***	H6
Self-rated health	←	Social engagement	0.303	0.073	8.691	***	H7

*, **, *** Indicate that the statistical results are significant at the confidence level of 10%, 5% and 1% respectively.

#### Mediating Effect of Social Engagement

The main methods for testing the mediating effect of social engagement are the causal step method and the Sobel method. However, the sample distribution of the mediating effect is not a normal distribution, which has raised questions about the reliability and effectiveness of these two methods. In recent years, three other methods have been proposed: the product distribution method, non-parametric bootstrap method and Markov chain Monte Carlo method (Penner et al., 2005). The bootstrap test proposed by Taylor et al. (2008) has a unique advantage in testing mediating effects in structural equation models. It not only allows variables to contain measurement errors but also utilizes all information in data, reducing the loss of information. Therefore, in this study, the mediating effect of social engagement was estimated by using the maximum likelihood method through bootstrapping with 5,000 replications under a 95% confidence interval. The estimated results are shown in Table 6. The confidence interval for the effect of the social engagement of elderly individuals on the path “social attitude → self-rated health” contained 0, while the confidence intervals for its effect on the paths “class identity → self-rated health” and “social relationship perception → self-rated health” did not contain 0. These results show that the mediating effect of social engagement among elderly individuals was significant for the paths “class identity → self-rated health” and “social relationship perception → self-rated health” but not for the path “social attitude → self-rated health.” Specifically, the mediating effect of social engagement among elderly individuals on the path “class identity → self-rated health” was 0.077, and the mediating effect on the path “social relationship perception → self-rated health” was 0.026.

**TABLE 6:** Mediating effect test results.

Mediating path	Indirect effect coefficient	Bootstrapping			
		Bias-corrected 95% CI		Unadjusted 95% CI	
		Lower	Upper	Lower	Upper
Social attitudes → Social engagement → Self-rated health	0.003	-0.014	0.021	-0.014	0.021
Class identity→ Social engagement → Self-rated health	0.077	0.056	0.103	0.055	0.101
Perception of social relations→ Social engagement → Self-rated health	0.026	0.009	0.046	0.008	0.045

## Discussion

Although people have linked social attitudes, class identity, and social relations with health, few people have tried to integrate these related factors and systematically analyze their impact on health. Previous studies have shown that in the special historical period of social transformation, people’s complex social psychology, which is composed of individual social attitudes, class identity and interpersonal perception of social relations, has an important impact on their health (Hans and Christian, 1996; Liu and Gu, 2014). Based on China’s representative national survey data and the rich connotation of social cognition theory, this study analyzes whether and how complex social cognition affects self-assessed health of the elderly in China and further provides a new theoretical and empirical basis for research on social cognition and elderly health.

Our results support our path hypothesis model. In general, the more positive the elderly’s social cognition is, the better their self-rated health status is. The positive perception of the three dimensions of social cognition (social attitude, class identity and social relationship perception) has a significant positive impact on the elderly’s self-rated health. These findings show that the government and society can stimulate positive changes in the elderly’s social attitude and class identity from the macro level of social trust and social justice, and can also stimulate positive changes in the elderly’s perception of social relations from the micro level of social engagement. These measures will bring benefits to the self-assessed health of the elderly. These are consistent with some conclusions of previous studies (Kim et al., 2014; Cheng et al., 2020). However, the difference is that current research overcomes previous incomplete research and analysis, and also clarifies the significant path of different dimensions of complex social psychology influencing self-rated health through path analysis.

We found that the higher the social trust perception of the elderly, the more positive their social attitude, and the higher the self-rated health level. At present, there are still some controversies about the impact of social trust on individual health. The main parties about the measurement of social trust. Social trust can be divided into special trust, general trust and universal trust. Special trust refers to the trust of one’s own family members, relatives or especially close friends and neighbors. General trust is the trust of the people you do not associate with very closely. Universal trust is the trust of people who are not in the same class as you, who are not familiar with you, and who are strangers. At present, a large number of studies have shown that social trust has a positive impact on individual self-reported health (Lin and Ensel, 1989; Skrabski et al., 2003; Cacioppo and Cacioppo, 2018). However, some studies have found that the elderly’s special trust in family has a significant positive impact on Chinese residents’ self-rated health status, while general trust has a significant negative impact (Poortinga, 2006). Different from previous studies, this paper uses a more macro universal trust to measure social trust, that is, the degree of trust of the elderly in whole society. We believe that even if the social resources are insufficient and the ability of the elderly to exchange resources with the society (the object of universal social trust) is limited, the elderly who have a higher sense of universal social trust have a high degree of trust in society and are more likely to get a sense of security and pride. Therefore they have sufficient confidence in subjective reporting their health, and provide higher health assessment.

We also found that the higher the elderly’s perception of social justice, the more positive their social attitude, and the higher their self-rated health level. This is consistent with the previous conclusion that social justice significantly affects the objective mental health of the elderly (Guo, 2017). We further found this conclusion in the level of elderly self-rated health, which provides further evidence for the comprehensive understanding and analysis of the influencing factors of elderly health. Among the 1,728 elderly people, nearly 68.9% had a subjective perception of social trust of 1–2 points, and 56.3% had a subjective perception of social justice of 1–2 points. This also fully shows that elderly individuals in China have a low degree of subjective perception of social trust and social justice, and the perception of social attitude is poor. Therefore, the government should regulate the social trust environment and improve the general trust level of the elderly to society by means of propaganda and guidance, policy restrictions, legal punishment and other means. At the same time, the government could formulate perfect policies, provide a social environment of loving and respecting the elderly, reduce social prejudice against the elderly, and improve the social attitude perception of the elderly.

In this study, we did not find the mediating role of social engagement in the “social attitude → self-rated health” path of the elderly. Further analysis found that the main reason is that the perception of social attitude of the elderly does not significantly affect their participation in social activities. Some studies have shown that there is a significant positive relationship between social trust and social engagement (Hu and Huang, 2019), and physical activity participation has a significant mediating role in the “social attitudes → self-rated health” pathway among Chinese residents (Du and Wang, 2020). We did not find evidence to support this conclusion in the elderly population. Our analysis suggests that this may be related to the special social engagement of the elderly. According to difference preface pattern theory, interpersonal network is a self-centered concentric circle, and the farther away from the center of the circle, the more distant the relationship. The theory of social escort and the theory of social emotional choice believe that as the elderly retire, their social network will weaken from outside to inside according to the relationship between closeness and distancing, and the frequency of their contact with members of the outermost network will gradually decrease or even be interrupted. Therefore, the elderly may participate in social interaction selectively on the basis of intimacy, positive emotional experience and familiarity with the field (Eriksson and Ng, 2015), and their perception of social trust and fairness may not affect the scope, degree and frequency of social interaction.

Our results also show that the elderly’s subjective class identity can directly predict the elderly’s self-rated health level, and can also indirectly affect the elderly’s self-rated health level through social engagement. Social engagement plays a mediating role between the elderly’s subjective class identity and self-rated health level. We found that each one-unit increase in class identity of the elderly can increase self-rated health levels by 0.077 through improvements in social engagement. And this indirect effect accounts for 31.2% of the total effect. That is to say, social engagement contributes 31.2% of the explanation of the influence of subjective class identity on self-rated health level of the elderly. This means that social engagement corresponding to class identity is an important factor to improve the self-rated health level of the elderly. Specifically, the higher the level of subjective class identification of the elderly, the higher the frequency of social engagement activities of the elderly, and the more active the elderly participate in social activities, the higher their self-evaluated health level, which further verifies that the conclusions of Eriksson et al. are also applicable to the elderly (Grundy and Sloggett, 2003; Zhou, 2010). In modern society, information technology plays a positive role in promoting individual’s subjective class identity (Shimada et al., 2014). Therefore, the government should actively distribute internet resources, effectively solve the technical difficulties of elderly individuals, and strive to improve the digital integration environment of elderly individuals to continuously improve their utilization rate of information technology and enhance their subjective class identity. At the same time, elderly individuals should strengthen their understanding of information technology, constantly learn new knowledge and ways of communication, and enrich their forms and contents of social engagement, and constantly cultivate and improve their enthusiasm for social engagement.

We also found that the social relationship perception of the elderly has a direct positive impact on their self-rated health level. At the same time, social engagement played a mediating role in the relationship between social relationship perception and self-reported health level. The estimated mediating effect was 0.026, accounting for 9.4% of the total effect. Specifically, the better elderly individuals’ perceptions of their social relations, the better their self-rated health. On the contrary, the stronger their feelings of lack of company, isolation and being left out, the lower their self-rated health level. Due to the aging of the elderly individuals and the atrophy of the social network, the elderly are easy to produce objective social isolation and subjective loneliness (Iecovich and Biderman, 2012). A large number of studies have shown that loneliness is common in the elderly (Hawkley et al., 2009), and it will seriously affect the physical and mental health of the elderly (Heikkinen and Kauppinen, 2004). Based on the basis of loneliness research and combined with the Chinese scene, we analyze the impact of social relations that may lead to the perception of loneliness on the self-rated health of the elderly. The conclusions proved the importance of social relationship perception on the health assessment of the elderly, and further gave the path and interventable measures to alleviate the loneliness of the elderly. On the one hand, we can start with the possible source of loneliness in the elderly to alleviate the generation of loneliness. Family members increase companionship to the elderly. The government and the community increase their attention to the elderly, reduce the lack of companionship, isolation and neglect of the elderly, thereby improving the self-rated health of the elderly. On the other hand, the government has established an environment for the elderly to actively participate in social activities through the introduction of corresponding policies and systems, and enhance the enthusiasm of the elderly in social engagement, thereby improving the self-evaluated health level of the elderly.

## Limitations

This study is subject to a number of limitations. First, the questionnaire measures social trust, lack of the measurement of general trust and special trust. The social network of the elderly will weaken from the outside to the inside according to the close relationship. Therefore, most of the social interaction of the elderly is the social interaction of acquaintances. In the future, we can consider the establishment of a social trust scale with more dimensions, and further research on the relationship between social attitudes and social engagement of the elderly. Second, we acknowledge that the excluded subsample of observations with missing data may not represent a random subset of the larger sample, which would reduce the accuracy of the estimation. Finally, as the study is based on a cross-sectional data set, no causal inferences are reported.

## Data Availability Statement

The datasets presented in this study can be found in online repositories. The names of the repository/repositories and accession number(s) can be found in Chinese General Social Survey, available at http://cnsda.ruc.edu.cn/index.php?r=projects/view&id=94525591.

## Author Contributions

YL contributed to the research design, methodology, data analysis, and draft preparation. YH contributed to literature review, problem analysis, and edited the manuscript. YN provided revised advice and participated in the revision of the manuscript. All authors contributed to the article and approved the submitted version.

## Conflict of Interest

The authors declare that the research was conducted in the absence of any commercial or financial relationships that could be construed as a potential conflict of interest.

## Publisher’s Note

All claims expressed in this article are solely those of the authors and do not necessarily represent those of their affiliated organizations, or those of the publisher, the editors and the reviewers. Any product that may be evaluated in this article, or claim that may be made by its manufacturer, is not guaranteed or endorsed by the publisher.
